# Jieduquyuziyin prescription for systemic lupus erythematosus: a system-level therapeutic strategy for immunological recalibration

**DOI:** 10.1186/s13020-026-01397-x

**Published:** 2026-04-14

**Authors:** Yihong Gan, Jingqun Liu, Shihui Zhou, Ke Lin, Xinchang Wang, Yongsheng Fan, Li Xu, Jie Bao

**Affiliations:** 1 The Second School of Clinical Medicine, Zhejiang Chinese Medical University, Hangzhou, 310053 Zhejiang China; 2 Department of Nephrology and Rheumatic Immunology, Tongde Hospital of Zhejiang Province, Hangzhou, 310000 Zhejiang China; 3 School of Basic Medical Sciences, Zhejiang Chinese Medical University, Hangzhou, 310053 Zhejiang China; 4 Department of Rheumatology, The Second Affiliated Hospital of Zhejiang Chinese Medical University, Hangzhou, 310005 Zhejiang China; 5 Jinhua Academy, Zhejiang Chinese Medical University, Jinhua, 321000 China

**Keywords:** Systemic lupus erythematosus, Traditional Chinese medicine, Jieduquyuziyin prescription, Chinese medicine formulae

## Abstract

**Background:**

Systemic Lupus Erythematosus (SLE) is a chronic autoimmune disease involving immune dysregulation, metabolic reprogramming, and multi-organ damage. The Jieduquyuziyin prescription (JP), a contemporary Chinese herbal formula based on the Traditional Chinese Medicine principle of “Jiedu Quyu Ziyin,” has shown clinical efficacy in SLE. This review aims to synthesize clinical and preclinical evidence explaining how JP achieves systemic therapeutic effects by targeting interconnected pathological pathways in SLE.

**Methods:**

We systematically searched PubMed, Web of Science, and CNKI (January 1999–December 2025) for clinical trials, preclinical studies, and multi-omics analyses investigating JP or its bioactive compounds in SLE.

**Results:**

Clinical evidence demonstrates that JP-containing regimens significantly reduce SLEDAI scores, alleviate lupus nephritis, lower glucocorticoid requirements, and improve quality of life in SLE patients. Preclinical studies in SLE-prone MRL/lpr mice reveal that JP targets key SLE pathogenic pathways: it restores Th17/Treg balance, suppresses SLE-associated pathogenic B cell proliferation via AKT/mTOR inhibition, and promotes apoptosis of double-negative T cells through UBE2M-mediated Bim upregulation. JP inhibits macrophage IRAK1/ NF-κB signaling, attenuates lupus nephritis by activating renal FXR to suppress TGF-β1/α-SMA-driven fibrosis, and mitigates oxidative damage via Nrf2 activation. Addressing SLE-associated immunometabolic dysfunction, JP reverses pathological glycolytic reprogramming in lymphocytes via AMPK signaling and restores mitochondrial function. Additionally, JP remodels gut microbiota in SLE models, enriching Akkermansia and modulating short-chain fatty acid and bile acid metabolism to reinforce immune homeostasis.

**Conclusion:**

This review validates JP as a system-level therapeutic strategy targeting immune dysregulation, metabolic reprogramming, and gut dysbiosis in SLE, supporting the TCM principle of “Jiedu Quyu Ziyin” and its integration with modern immunology.

**Graphical abstract:**

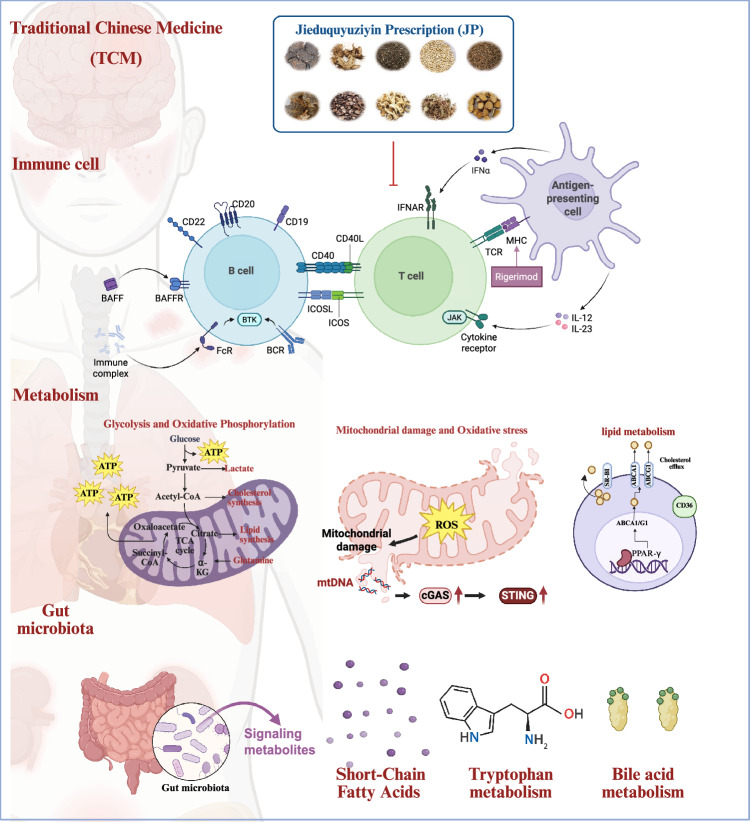

## Introduction

Systemic lupus erythematosus (SLE) is a chronic systemic autoimmune disease characterized by a loss of immune tolerance, production of pathogenic autoantibodies, and immune complex formation, which collectively drive progressive inflammatory injury across multiple organs and systems [1]. The clinical presentation of SLE is highly heterogeneous, ranging from common manifestations such as arthritis (affecting approximately 90% of patients) [2] and cutaneous lesions (60–80% of patients) [3] to severe, life-threatening complications. Among these, lupus nephritis (LN), neuropsychiatric SLE (NPSLE), and cardiovascular disease (CVD) represent leading causes of mortality. LN develops in approximately 40% of SLE patients, with about 10% progressing to end-stage renal disease within 10 years, establishing it as a major contributor to SLE-related deaths [4]. NPSLE encompasses a spectrum of conditions, from non-specific symptoms like headache and cognitive dysfunction to severe syndromes such as Guillain–Barré syndrome, with associated mortality second only to LN [5]. Furthermore, chronic inflammation and immune dysregulation promote premature vascular injury and accelerated atherosclerosis in SLE patients, rendering CVD a key determinant of long-term mortality [6]. Overall, the heterogeneity in clinical manifestations, unpredictable disease course, and individual variability in treatment response constitute major challenges in SLE management and contribute to a substantial disease burden, particularly among women of reproductive age, who account for approximately 90% of cases [7, 8].

The pathogenesis of SLE arises from complex interactions among genetic susceptibility, environmental triggers, and immune system dysregulation, leading to a breakdown of self-tolerance and persistent autoimmunity [9]. Genetically, genome-wide association studies have identified numerous SLE-risk loci enriched in key pathways: (1) type I interferon signaling, a hallmark of SLE, involving genes such as *TLR7* and *IRF5*; (2) antigen presentation and immune cell activation, particularly mediated by MHC class II (e.g., HLA-DRB1); and (3) clearance of apoptotic debris and immune complexes, where deficiencies in complement components (e.g., C1q, C4A) lead to accumulation of self-antigens [5, 9]. Environmentally, ultraviolet radiation can trigger disease by inducing apoptosis and exposing self-antigens [8]; infections such as Epstein–Barr virus may break tolerance via molecular mimicry [10]; and factors including sex hormones (e.g., estrogen) [11], smoking [12], and certain drugs [13] also contribute. These environmental exposures act on a permissive genetic background, activating central pathogenic pathways: dysregulated plasmacytoid dendritic cells drive type I interferon overproduction, a core molecular feature of SLE [14]; autoreactive B cells—supported by excess BAFF signaling and T cell help—differentiate into antibody-producing plasma cells, while impaired regulatory T cell function and heightened helper T cell activity lead to adaptive immune imbalance [15]. The resulting autoantibodies form immune complexes that deposit in tissues, activate complement, and promote chronic inflammation, ultimately causing multi-organ damage.

In SLE pathogenesis, both innate and adaptive immune abnormalities interact to shape a pro-inflammatory microenvironment. In the adaptive arm, dysregulated B cell development, activation, and differentiation lead to autoantibody production and immune complex deposition, which instigate complement activation and tissue injury [16]. Altered B cell subset differentiation influences disease outcomes: although memory B cells in SLE show attenuated BCR signaling, they remain hyper-responsive to Toll-like receptor (TLR) activation, type I interferon signaling, and CD40–CD154-mediated T cell help, a functional bias that may sustain autoimmunity [17]. TLR7/9 activation, along with cytokines such as IFN-γ and IL-21, promotes the expansion and differentiation of age-associated B cells, which contribute to SLE pathogenesis [18]. Among T cells, CD4⁺ helper T cells and CD8⁺ cytotoxic T cells play major roles, with emerging evidence implicating rare subsets such as double-negative T cells [19]. CD4⁺ T cells differentiate into functionally distinct subsets—Th1, Th2, Th17, and regulatory T cells (Tregs). Th1 and Th17 cells promote inflammation, whereas Th2 cells and Tregs exert immunoregulatory effects [20]. An elevated Th17/Treg ratio is a prominent immunologic abnormality in SLE, leading to excessive IL-17 production and aggravated tissue inflammation [21]. Th1/Th2 imbalance is also a key pathogenic mechanism, with Th1-derived cytokines (e.g., IL-1β, TNF-α, IFN-γ) correlating with disease severity and exhibiting biomarker potential [22], while Th2-associated cytokines (e.g., IL-4, IL-10, IL-6, TGF-β) modulate humoral immune responses [23]**.** In the innate immune compartment, myeloid cells are deeply involved in SLE [24]. Monocytes, macrophages, and tissue-resident macrophages contribute to inflammation and tissue injury through polarization shifts (e.g., M1/M2 imbalance) [25], metabolic and epigenetic reprogramming [26], and transcriptional regulation [27]. Activated neutrophils release neutrophil extracellular traps (NETs), which enhance local and systemic inflammation, provide autoantigens, and activate plasmacytoid dendritic cells, thereby reinforcing type I interferon production and contributing to disease chronicity [28, 29]. Together, immune cells and cytokines form a complex, dysregulated network in SLE. Given the central role of immunopathology, targeting abnormal immune activation and inflammatory pathways has become a key therapeutic strategy.

In the field of therapeutics, the management of SLE has traditionally depended on the use of glucocorticoids in conjunction with broad-spectrum immunosuppressants, including hydroxychloroquine, mycophenolate mofetil, and cyclophosphamide. This treatment paradigm has notably enhanced overall survival rates [30]. Nevertheless, these conventional therapies are frequently constrained by significant adverse effects, such as an elevated risk of opportunistic infections [31], metabolic disturbances [32], reproductive toxicity [20], and cumulative organ damage [33]. These issues contribute to a clinical challenge characterized by suboptimal efficacy and treatment-related harm [34]. Recent advancements in the understanding of SLE immunopathology have facilitated the development of targeted biologic agents, such as belimumab and rituximab, which target B cells and T cell interactions [35], as well as anifrolumab, which inhibits the type I interferon receptor [36]. This progress signifies a shift towards precision medicine. Notably, chimeric antigen receptor (CAR) T-cell therapy has demonstrated promising outcomes in cases of refractory SLE, with several studies documenting profound clinical remission and manageable adverse effects, primarily mild cytokine release syndrome [37, 38]. Despite these advancements, the novel therapies encounter several limitations, including incomplete response rates, uncertain long-term safety, and high costs, which constrain their widespread adoption. Consequently, there is an urgent need to develop more effective, accessible, and precisely targeted treatments.

Within this framework, traditional Chinese medicine (TCM), supported by its extensive historical application and holistic theoretical foundation, offers a complementary perspective for addressing the clinical heterogeneity of SLE. In TCM theory, SLE is often classified under concepts such as “Yin Yang Du,” with its core pathogenesis believed to involve Yin deficiency, blood stasis, and the accumulation of heat toxins. The therapeutic principle of “clearing toxins, resolving stasis, and nourishing Yin” (Jiedu Quyu Ziyin) underpins the use of the Jieduquyuziyin prescription (JP), a modern formula developed by Professor Fan Yongsheng through decades of clinical observation and systematic research, with the aim of enhancing therapeutic efficacy while mitigating the adverse effects associated with glucocorticoid therapy. JP represents Professor Fan Yongsheng’s effort to bridge traditional TCM concepts with contemporary pathophysiological understanding of SLE, seeking to translate these theoretical principles into a targeted therapeutic strategy: “clearing toxins” (Jiedu) corresponds to suppressing the hyperactive immune responses characteristic of SLE, eliminating circulating autoantibodies and inflammatory mediators such as interferons and interleukins, thereby exerting anti-inflammatory and immunomodulatory effects that alleviate “heat-toxin” manifestations including cutaneous erythema and arthritis; “resolving stasis” (Quyu) aims to ameliorate the hypercoagulable state and microcirculatory disturbances prevalent in SLE patients, preventing vascular endothelial injury and thrombosis—a therapeutic strategy that aligns with modern approaches of anticoagulation and microcirculatory improvement to delay organ fibrosis, particularly in the kidney; and “nourishing Yin” (Ziyin) serves to protect glandular secretory function, alleviating symptoms such as xerostomia and xerophthalmia, and more importantly, restores internal environmental homeostasis by modulating the hypothalamic–pituitary–adrenal axis while enhancing cellular antioxidant capacity, thereby counteracting the adverse effects of glucocorticoid therapy and repairing the cumulative tissue damage resulting from chronic consumptive disease processes. Thus, the integrated principle of “Jiedu Quyu Ziyin” represents a system-level therapeutic strategy that concurrently targets three interconnected pathological dimensions in SLE—excessive inflammation and immune activation (toxins), microcirculatory disturbances and fibrotic progression (stasis), and cellular/tissue damage with impaired regenerative capacity (Yin deficiency)—an approach that aligns with modern systems biology perspectives on SLE as a complex disease requiring interventions that address multiple dysfunctional pathways simultaneously. Research indicates that JP exerts multi-target effects by modulating T cells, B cells, and monocytes/macrophages, thereby ameliorating SLE and severe complications such as LN [39–41]. Active components derived from the formula, including alisol A, luteolin, and apigenin, have demonstrated efficacy in alleviating core disease manifestations and mitigating treatment-related side effects [42–44]. Clinical evidence indicates that integrated traditional Chinese and Western medicine approaches may offer synergistic benefits, including better disease control, reduced glucocorticoid dosage, lower infection risk, and delayed organ damage.

**Fig. 1:**
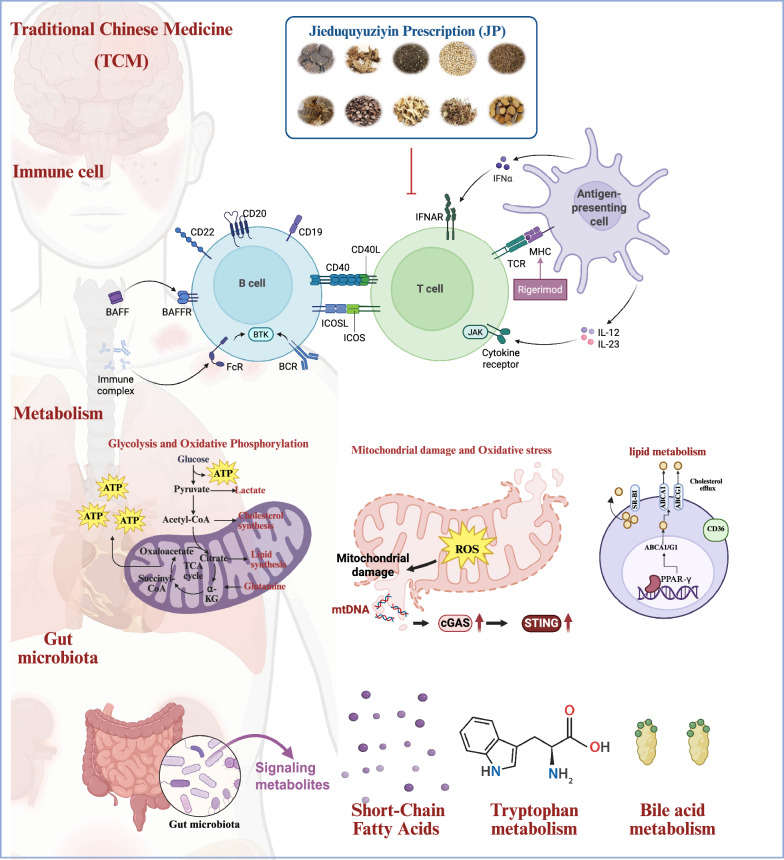
Mechanisms of the Jieduquyuziyin prescription (JP) in the treatment of systemic lupus erythematosus (SLE). This schematic illustrates the multi-system mechanisms through which JP, a representative TCM prescription, exerts its therapeutic effects on SLE. The model proposes that JP acts concurrently on three core pathological aspects: (1) Immunomodulation: Normalizing the function and balance of dysregulated immune cells, including T cells, B cells, and antigen-presenting cells. (2) Metabolic Reprogramming: Correcting aberrant energy metabolism in immune cells. (3) Gut Microbiota & Metabolism: Modulating gut microbiota composition and its associated metabolic activities, particularly the production of short-chain fatty acids (SCFAs) and the metabolism of tryptophan and bile acids. The synergistic interplay among these pathways is shown to collectively contribute to the alleviation of inflammation and tissue damage in SLE

To enhance the integration of TCM into contemporary medical practice, it is crucial to elucidate its mechanisms through modern scientific methodologies. To the best of our knowledge, no comprehensive review has systematically summarized the mechanisms by which TCM formulations or natural compounds derived from medicinal plants exert protective or therapeutic effects in SLE. This review aims to synthesize scientific evidence on how TCM may restore immune homeostasis, provide direct organ protection, and influence metabolic reprogramming in SLE. Particular emphasis will be placed on the emerging “gut–microbiota–metabolism–immunity” axis as a potential mechanism through which the TCM systemically recalibrates immune balance. By employing systems biology tools such as network pharmacology and multi-omics integration, we seek to translate the holistic concepts of TCM into testable molecular network models, thereby advancing the modernization of TCM in the treatment of SLE **(**Fig. 1**).**

## Evidence for the clinical efficacy of Chinese medicine in SLE

The therapeutic application of TCM in the management of SLE is increasingly substantiated by contemporary clinical research. Employed as a complement to conventional Western medicine (WM), TCM seeks to augment therapeutic efficacy while minimizing adverse effects, a strategy often referred to as “enhancing efficacy and reducing toxicity.” Clinical evidence indicates that this integrative approach may enhance disease remission rates, reduce SLE Disease Activity Index (SLEDAI) scores, modulate specific autoantibody levels, and enable glucocorticoid tapering while alleviating associated side effects. Notable clinical trials, such as those examining the JP, provide corroborative evidence for the potential of TCM to improve outcomes for SLE patients, both as a standalone treatment and in combination with other therapies (Table 1). These clinical findings lay the groundwork for further exploration into the mechanistic underpinnings of TCM’s effects in SLE.

**Table 1:** Clinical efficacies of TCM for SLE treatment

No	Name/composition	Method	Clinical manifestation	References
1	Shenqi Dihuang decoction	Meta-analysis	For lupus nephritis, the SQD formulation was superior to Western medicine alone, demonstrating improvements not only in laboratory and disease activity markers (Complement C3, ESR, SLEDAI) but also in key clinical outcomes, including renal function preservation, overall lupus activity, and reduced infection risk	[45]
2	Jieduquyuziyin prescription	RCT	Integrated therapy with the Jieduquyuziyin prescription and Western medicine offers comprehensive benefits for SLE patients, including significant alleviation of clinical manifestations, reduction in disease activity, improved NLR and PLR levels, and a favorable safety profile	[46]
3	Xin Gan Bao Capsule; Jin Shui Bao Capsule; Huang Kui Capsule	Meta-analysis	Xin Gan Bao Capsule has shown good efficacy in improving efficiency and the level of complement C3, as well as lowering 24-h urine protein. Jin Shui Bao Capsule and Huang Kui Capsule have a good efficacy in treating lupus nephritis (LN)	[47]
4	1. Those tonifying the kidney (KF) group, 2. Those activating blood circulation (BF) group, 3. the “other TCM therapy” group	RCT	This cohort study identified a protective effect of TCM against pneumonia in SLE patients, with optimal regimens comprising KF formulae for over 90 days and BF formulae for under 30 days	[48]
5	Zhibai Dihuang pill (ZBDH)	Meta-analysis	Modified ZBDH enabled SLE patients to reduce steroid doses while maintaining low disease activity, with a favorable tolerability profile	[50]
6	Jieduquyuziyin prescription	RCT	The integrated traditional Chinese medicine group demonstrated significant improvements in specific lipid parameters (TC and LPa) compared to the Western medicine group, while the Jieduquyuziyin prescription showed potential in preventing and managing steroid-induced dyslipidemia in SLE patients	[51]
7	Curcumin and Curcuma longa Extract	Meta-analysis	Curcumin was well-tolerated and associated with numerical improvements in SLEDAI scores and reductions in IL-6 and TGF-β1 levels, although these changes did not reach statistical significance	[52]
8	Qing Shen Fang	RCT	The addition of Qing Shen Fang to conventional immunosuppressants (cyclophosphamide and prednisolone) resulted in clinical improvement in patients with lupus nephritis, concurrent with significant reductions in serum cytokines IFN-γ and IL-4	[53]
9	Dan‐Chi‐Liu‐Wei combination (DCLWC)	RCT	In patients with SLE, adjunct DCLWC was safe and associated with a potential reduction in disease activity, but failed to exhibit a steroid-sparing effect after 6 months of treatment	[54]
10	Raw astragalus 50 g, Duhua 15 g, Chuanqi 15 g, Astragalus membranaceus, Salvia miltiorrhiza and Chinese yam 20 g each	RCT	The combined treatment of traditional Chinese and Western medicine can successfully prevent the secretion of serum IL-6, IL-18, and TNF-α, control the development of disease, boost the therapeutic outcome, and alleviate the immune injury of the body	[55]
11	Chinese medicine therapy for activating blood and dredging collaterals (ABDC)	RCT	Chinese medicine ABDC therapy could effectively alleviate clinical symptoms and improve joint function of patients with SLE complicated with avascular necrosis of the femoral head	[56]
12	Total glucosides of paeony	Meta-analysis	The combined use of TGP can enhance the clinical efficacy of SLE without increasing the incidence of adverse effects	[57]
13	Dan Bai Xiao Formula (DBXF)	RCT	The DBXF-containing regimen conferred multiple advantages over standard therapy in lupus nephritis, including faster resolution of proteinuria/hematuria, smoother steroid reduction, fewer methylprednisolone pulses, and a superior safety profile	[58]
14	Cicimifuga rhizome 9 g, Oldenlandia herb 18 g, Southernwood 15 g, Red peony root 12 g, Moutan bark 12 g, Rehmannia root 15 g, and Turtle shell 12 g	RCT	After 6 months of treatment, the integrated medicine group demonstrated significant improvements in lipid profiles compared to the Western medicine group in SLE patients with corticosteroid-induced hyperlipidemia—including reduced levels of TC, TG, LDL-C, and VLDL-C, along with elevated levels of HDL-C and ApoA (*P* < 0.05). These improvements were sustained during the follow-up period	[59]
15	Huaiqihuang granules	RCT	The adjuvant treatment by Huaiqihuang granules can effectively reduce the inflammatory response, decrease the disease activity of SLE, and lower the recurrence rate in children with SLE relapse	[60]
16	Langchuang No.1 or 2	RCT	Following treatment, both the integrated medicine and Western medicine groups showed increased Th1/Th2 and Tc1/Tc2 ratios (*P* < 0.05 and *P* < 0.01). However, the increase was significantly more pronounced in the integrated medicine group compared to the Western medicine-only group (*P* < 0.05)	[61]
17	Jieduquyuziyin prescription	RCT	The SF-36 scale shows good reliability and validity in assessing quality of life in SLE patients. Integrated therapy with Jieduquyuziyin prescription and corticosteroids demonstrated superior improvement in SF-36 scores across all domains and total scores at both assessments compared to Western medicine alone	[62]
18	Jieduquyuziyin prescription	RCT	Jieduquyuziyin prescription, together with prednisone, can more effectively suppress the expression of Fas in T lymphocyte subsets in SLE patients and improve the disturbed immunological function	[63]
19	Jieduquyuziyin prescription	RCT	Administration of the Jieduquyuziyin prescription may help correct the characteristic sex hormone dysregulation observed in patients with systemic lupus erythematosus	[64]
20	Jieduquyuziyin prescription	RCT	After the second treatment course, the Chinese medicine group showed significant differences in active/inactive disease distribution (*P* < 0.05) and greater reduction in prednisone dosage (*P* < 0.01) versus controls, demonstrating the therapy’s dual benefit in controlling SLE activity and facilitating steroid withdrawal	[65]

### Disease activity and laboratory parameters

Clinical research indicates that TCM formulations, which are based on the principles of “detoxification, removing stasis, and nourishing Yin,” particularly the JP, can effectively reduce global disease activity in patients with SLE. This efficacy is evidenced by significant improvements in standardized assessment tools, such as the SLEDAI. Integrated therapy combining TCM and WM has demonstrated superior efficacy compared to WM alone in modulating key immunological biomarkers. A meta-analysis of 14 randomized controlled trials (n = 1002) revealed that the Shenqi Dihuang decoction, when used in conjunction with conventional WM, significantly reduced anti-double-stranded DNA (anti-dsDNA) antibody titers and erythrocyte sedimentation rate, while enhancing complement C3 and C4 levels. This combination therapy effectively slowed the progression of lupus nephritis and exhibited a favorable safety profile [45]. Furthermore, specific herbal combinations have been correlated with improvements in systemic inflammatory markers, including the neutrophil-to-lymphocyte ratio (NLR) and platelet-to-lymphocyte ratio (PLR) [46].

### Complication management and organ protection

TCM has demonstrated beneficial effects in the management of complications associated with SLE, particularly lupus nephritis. The adjunctive use of Chinese patent medicines—such as Xin Gan Bao Capsule, Jin Shui Bao Capsule, and Huang Kui Capsule—in conjunction with standard immunosuppressive regimens has been correlated with enhanced clinical efficacy, improved TCM syndrome scores, and accelerated resolution of hematuria and proteinuria [47]. Beyond renal protection, population-based cohort studies suggest that TCM use is associated with a significantly reduced risk of serious infections, such as pneumonia, in SLE patients. This finding is particularly relevant given the immunosuppressive nature of conventional treatments [48].

### Steroid-sparing effects and side effect management

A notable advantage of integrating TCM with WM therapy is its potential to spare the use of steroids, thereby reducing glucocorticoid-related toxicities [49]. Clinical data indicate that patients receiving TCM adjunct therapy can achieve more rapid and stable tapering of glucocorticoids while maintaining low disease activity [50]. Additionally, TCM interventions have demonstrated efficacy in alleviating corticosteroid-induced metabolic complications, such as hyperlipidemia, by significantly modulating serum lipid parameters, including total cholesterol, triglycerides, LDL-C, and lipoprotein(a) [51]. In conclusion, the integration of TCM into contemporary management of SLE represents a promising strategy for achieving more comprehensive disease control, reducing glucocorticoid dependence, and enhancing quality of life. Further research is warranted to elucidate the molecular mechanisms underlying these clinical effects and to optimize treatment strategies.

## Traditional Chinese medicine in SLE: a systemic remodeling of immunity from innate to adaptive responses

Mechanisms The immunopathogenesis of SLE is characterized by a complex dysregulation of both innate and adaptive immune systems [66]. This intricate dysregulation is evidenced by abnormal autoimmune responses, which are driven by the coordinated activities of T-cells and B-cells, alongside the atypical activation of monocytes, macrophages, and neutrophils. These interconnected immunological disturbances collectively establish a self-perpetuating cycle of autoimmune activation, thereby facilitating the initiation and progression of the disease. Emerging preclinical evidence from well-established SLE models indicates that Chinese herbal formulations and their active constituents exhibit multi-target immunomodulatory effects. A notably well-documented example is the JP, developed by Professor Fan Yongsheng through decades of clinical observation and systematic research. This formula is grounded in the TCM pattern diagnosis of “heat-toxin exuberance, yin deficiency, and blood stasis,” which is frequently observed in SLE patients. It was specifically designed to enhance therapeutic efficacy while mitigating the side effects associated with glucocorticoid use.

### Calibration of innate immunity

The innate immune system plays a pivotal role in the initiation and perpetuation of SLE, characterized by its intricate network of antigen-presenting cells, complement cascades, and a multitude of pattern recognition receptors that are instrumental in disrupting self-tolerance and sustaining chronic inflammation [67]. Macrophages, as essential components of the innate immune response, are actively involved in the progression of SLE through various mechanisms. In the context of lupus nephritis, renal macrophages that express Fcγ receptors respond to deposited IgG immune complexes, with tissue-resident macrophages identified as the primary responders to these complexes [24]. The polarization state of these macrophages is a critical determinant of disease outcomes, rendering macrophage polarization a promising therapeutic target in SLE [68].

TCM components modulate various aspects of innate immunity through sophisticated mechanisms. JP suppresses peritoneal macrophage activation in MRL/lpr mice through inhibition of the IRAK1-Nuclear Factor kappa-B (NF-κB) pathway [69] and similarly inhibits inflammatory activity in bone marrow-derived macrophages via the same mechanism [70]. This targeted approach reduces the production of pro-inflammatory cytokines while maintaining essential macrophage functions.

Qinghao Biejia decoction, a foundational formula of JP, enhances M2 macrophage polarization by regulating ABCA1/G1-mediated cholesterol efflux, thereby reprogramming macrophages toward a tissue-reparative phenotype and improving SLE manifestations [71]. Additionally, luteolin, present in multiple Chinese herbs, attenuates macrophage infiltration-related renal injury by suppressing HIF-1α expression and oxidative stress, addressing the hypoxic microenvironment in inflamed tissues [44].

**Fig. 2:**
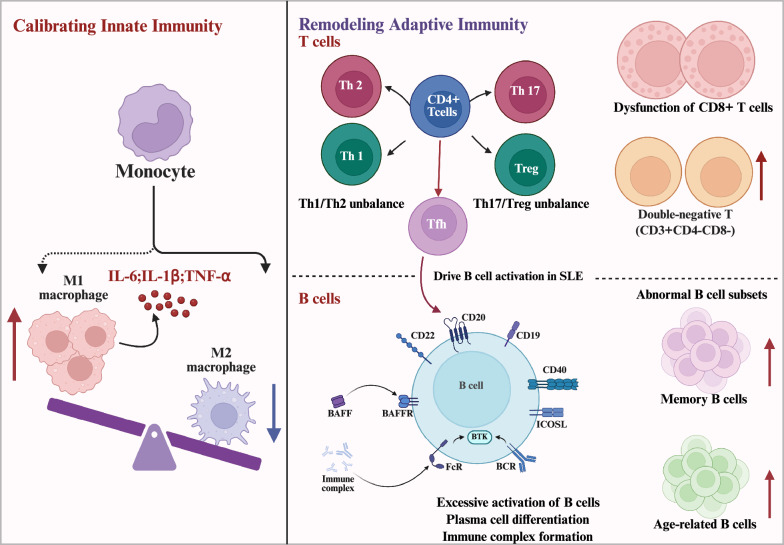
Modulation of adaptive and innate immunity by Jieduquyuziyin prescription (JP) in SLE. Schematic diagram illustrating the key immune cell subsets targeted by JP to regulate adaptive immunity and calibrate innate immune responses. The prescription acts on lymphocytes, including CD4⁺ T cell subsets such as Th1, Th2, Th17, Treg, and follicular helper T cells (Tfh), as well as double-negative T cells and B cells (memory B cells, age-associated B cells), to restore immune balance. Tfh cells play a critical role in providing help to B cells within germinal centers, and their modulation by JP contributes to the suppression of pathogenic autoantibody production. The prescription also modulates innate immune cells, including monocytes and macrophages (M1/M2 polarization), contributing to the overall amelioration of SLE immunopathology

Beyond macrophage regulation, emerging evidence suggests TCM components also influence neutrophil extracellular trap formation, dendritic cell maturation, and natural killer cell function, representing a comprehensive approach to innate immune calibration in SLE** (**Fig. 2**).**

### Remodeling adaptive immunity

#### T-cell regulation and rebalancing

Adaptive immune dysregulation represents a fundamental aspect of SLE pathogenesis, characterized by intricate T-cell and B-cell dysfunction. The abnormal CD4⁺ T-cell subset distribution, particularly the significantly elevated Th17/Treg ratio and excessive T follicular helper cell activation, creates a pro-inflammatory microenvironment that provides sustained aberrant help to B cells. This T-cell imbalance further amplifies autoimmune responses through multiple cytokine networks and cell–cell interactions. The expanded populations of double-negative T cells (CD3⁺CD4⁻CD8⁻) in SLE patients represent another layer of complexity, highlighting the involvement of unconventional T-cell subsets in disease pathogenesis [72].

JP demonstrates comprehensive effects on T-cell homeostasis through multiple interconnected mechanisms. The prescription ameliorates SLE symptoms by reprogramming CD4⁺ T-cell metabolism, primarily through inhibition of glycolytic pathways via the AMP-Activated Protein Kinase (AMPK)/Mammalian Target of Rapamycin (mTOR) signaling axis, thereby effectively restoring Th17/Treg balance [73]. This metabolic reprogramming represents a sophisticated approach to immune regulation, targeting the fundamental bioenergetic requirements of activated autoimmune cells.

At the transcriptional level, JP suppresses Th17 proliferation and IL-17 production in MRL/lpr mice through coordinated downregulation of CaMK4 at both transcriptional and translational levels, revealing another precise mechanism underlying its clinical application [41]. The CaMK4 pathway has been identified as a crucial regulator of IL-17 production, making this finding particularly relevant for SLE pathogenesis.

Addressing the well-established DNA hypomethylation in SLE CD4⁺ T-cells [74], JP treatment demonstrates additional protective effects in MRL/lpr mice by attenuating renal pathology and enhancing CD11a and CD70 methylation through the miR-29b-Sp1/DNMT1 axis [75]. This epigenetic regulation represents a sophisticated layer of immunomodulation, potentially reversing fundamental abnormalities in SLE T cells. Notably, JP upregulates global DNA methylation and MeCP2 expression in CD4⁺ T-cells to an extent comparable to prednisone acetate, suggesting significant potential for epigenetic modulation in SLE management while avoiding steroid-related side effects [76].

Regarding the pathological double-negative T-cell populations, JP restores cellular homeostasis in SLE by suppressing UBE2M expression. This reduction in the ubiquitin-conjugating enzyme decreases Bim ubiquitination and degradation, thereby promoting Double-negative T-cell (DNT)apoptosis and ameliorating lupus phenotypes through restoration of normal apoptotic pathways [39]. Complementary evidence shows that apigenin, a dietary flavonoid present in many Chinese herbs, induces CD8⁺ T-cell apoptosis and suppresses macrophage recruitment through STAT3/IL-17 pathway inhibition, suggesting additional therapeutic avenues for modulating T-cell responses in SLE patients [43] **(**Fig. 2**).**

#### B-cell modulation and antibody production

The B-cell compartment in SLE demonstrates equally complex dysregulation. Autoreactive B cells undergo BAFF-mediated activation and differentiation into antibody-producing plasma cells, generating pathogenic autoantibodies against nuclear antigens. The resulting immune complexes deposit in tissues, activate complement cascades, and drive chronic inflammatory organ damage through Fc receptor engagement and neutrophil activation [77]. Further complexity arises from abnormal differentiation of specialized B-cell subsets, including double-negative B cells, memory B cells, and age-associated B cells, all contributing to SLE progression through distinct mechanisms [78].

JP exerts multi-faceted control over B-cell pathology through several coordinated mechanisms. The prescription inhibits Protein Kinase B (AKT)/mTOR/c-Myc signaling to suppress B-cell proliferation and activation [79], while simultaneously restraining glycolysis-dependent B-cell activation through AMPK/PKM2 pathway stimulation [80]. This dual approach targets both the fundamental signaling pathways and metabolic requirements of hyperactive B cells in SLE.

Further research reveals that JP treatment reduces integrin αV expression in B cells from SLE patients and mice, ameliorates renal pathology, and diminishes age-associated B cells in extrafollicular regions by modulating ZEB2/ITGAV-expressing effector B cells [41]. These coordinated actions attenuate tissue inflammation and autoantibody production, demonstrating the comprehensive immunomodulatory capacity of TCM formulations in addressing the complex B-cell abnormalities in SLE **(**Fig. 2**).**

### Targeting key pathogenic signaling pathways

The type I interferon signature represents a core pathogenic feature in SLE, with interferon-regulated epigenomic landscapes influencing multiple gene sets, including MAPK, Phosphoinositide 3-Kinase (PI3K)/AKT, Nuclear Factor Erythroid 2-Related Factor 2 (Nrf2), TLR families, and NF-κB targets [81]. Whole-exome sequencing of lupus nephritis patients further reveals disease-associated variants in NF-κB, interferon I, PI3K/AKT, JAK/STAT, RAS/MAPK, and complement pathways [82], highlighting the complex signaling network underlying SLE pathogenesis. Traditional Chinese Medicine components target these multiple signaling cascades to restore immune homeostasis through coordinated modulation.

#### TLR and NF-κB pathways

Given the established association between TLR polymorphisms and SLE, and TLR-mediated control of effector B-cell differentiation [83], JP suppresses disease progression in pristane-induced ApoE⁻/⁻ SLE mice with atherosclerosis through TLR9/MyD88 inhibition and cholesterol efflux promotion [40]. Additional research shows that JP alleviates SLE by adjusting the ERα-miR146a-TLR7 circuit, revealing novel therapeutic mechanisms that integrate nuclear receptor signaling with innate immune regulation [84].

NF-κB hyperactivation drives aberrant immunity and chronic inflammation in SLE, making it a prime therapeutic target. JP inhibits macrophage activation through IRAK1-NF-κB signaling [69, 70]**,** and ameliorates lupus nephritis through FXR activation with concurrent NF-κB and α-SMA suppression [85]. Resveratrol contributes additional regulation by upregulating FcγRIIB via Sirt1-mediated p65 deacetylation, selectively reducing B-cell numbers, autoantibody production, and renal pathology [86].

#### Metabolic and stress response pathways

The PI3K/AKT axis represents another key target, with its dual role in both activating and constraining immune responses in SLE [87]. JP reduces glucocorticoid side effects by suppressing hepatic gluconeogenesis through PI3K/Akt/PGC-1α signaling [88], demonstrating the interconnectedness of metabolic and immune pathways. Other natural compounds show complementary effects: phlorizin drives FOXP3-dependent Treg differentiation via PI3K/AKT activation [89], while curcumin attenuates renal neutrophil infiltration and proinflammatory cytokine release through PI3K/AKT/NF-κB inhibition [90].

Nrf2, a central antioxidant regulator, provides further therapeutic opportunities in SLE by counteracting oxidative stress, a significant contributor to tissue damage [91, 92]. JP ameliorates lupus nephritis by enhancing renal Nrf2 expression and suppressing oxidative stress, thereby complementing prednisone effects and reducing steroid-related toxicity [93]. Other compounds employ similar pathways: esculetin alleviates murine lupus nephritis through complement inhibition and Nrf2 activation [94], while taxifolin activates Nrf2 to suppress NETosis in lupus and antiphospholipid syndrome models [95].

AMPK signaling integrates metabolic and immune responses in SLE, with metformin already demonstrating adjunct benefits in SLE treatment through AMPK activation [96]. JP contributes to this regulatory network through multiple mechanisms: it inhibits glycolysis-dependent B-cell activation via AMPK/PKM2 stimulation [80] and restores Th17/Treg balance through AMPK/mTOR modulation [73]. Piperine further expands these options by suppressing NLRP3 inflammasome activation and pyroptosis in tubular epithelial cells via AMPK targeting, thereby mitigating LN progression through regulation of inflammatory cell death pathways [97].

**Fig. 3:**
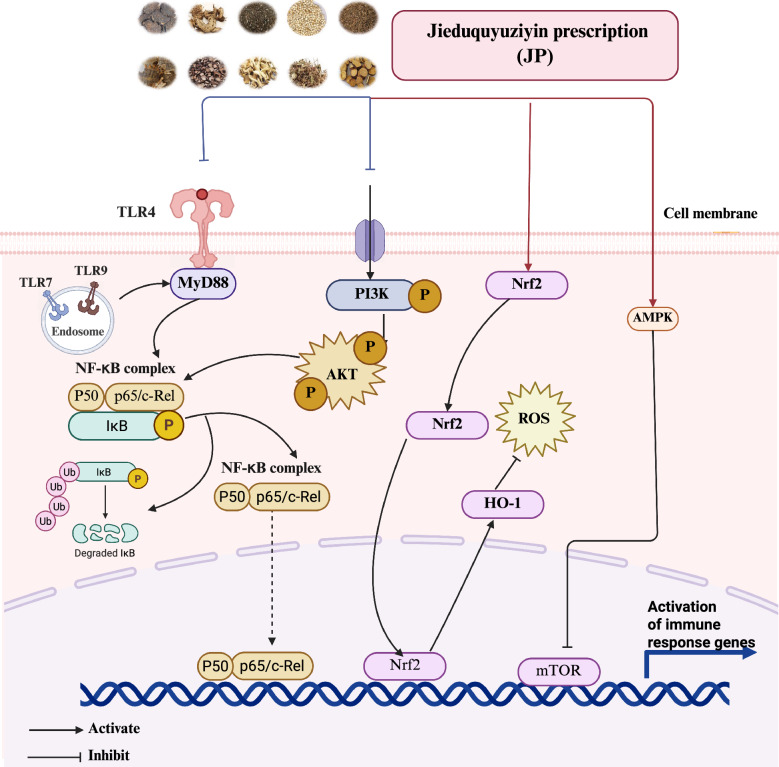
Multi-target signaling mechanisms of Jieduquyuziyin prescription (JP) in SLE intervention. This model illustrates how JP coordinately regulates key signaling pathways to exert therapeutic effects in SLE. JP targets both cell surface receptor TLR4 and endosomal Toll-like receptors (TLR7 and TLR9) localized within intracellular endosomes. By modulating these receptors and their downstream MyD88 signaling, JP attenuates NF-κB pathway activation and subsequent pro-inflammatory gene transcription. Additionally, JP influences the PI3K/AKT/mTOR pathway, enhances the Nrf2 antioxidant response to alleviate oxidative stress (ROS), and modulates the AMPK pathway. The concerted action on these pathways—inhibiting inflammatory NF-κB, enhancing antioxidant Nrf2, and correcting metabolic dysfunction—contributes to the overall amelioration of the aberrant immune and metabolic environment in SLE

The accumulated evidence demonstrates that Traditional Chinese Medicine exerts its therapeutic effects in SLE through sophisticated multi-level immunomodulation. By simultaneously targeting adaptive immunity (through T-cell and B-cell regulation), calibrating innate immune responses (via macrophage polarization and NETosis suppression), and intervening in critical signaling pathways (including NF-κB, TLR, PI3K/AKT, and AMPK cascades), TCM formulations create an integrated regulatory network that addresses the complexity of SLE pathogenesis. This multi-target approach represents a distinct advantage over single-target biologic therapies, potentially explaining the long-observed clinical benefits of TCM in SLE management **(**Fig. 3**).**

However, the therapeutic implications of these immunomodulatory effects extend beyond conventional immune pathways. Emerging research reveals that immune cell function and differentiation are fundamentally governed by cellular metabolic reprogramming and that SLE pathogenesis involves significant alterations in various metabolic pathways. The metabolic control of immune cell fate and function represents a crucial layer of regulation in autoimmune diseases, suggesting that the immunomodulatory capacity of TCM may be intrinsically linked to its influence on cellular metabolism. This intersection between immunology and metabolism provides a compelling framework for understanding the comprehensive therapeutic effects of TCM in SLE and forms the basis for our subsequent exploration of metabolic reprogramming in this autoimmune disorder.

## Organ protection and metabolic regulation: emerging mechanisms of TCM in SLE management

The evolving field of immunometabolism has fundamentally transformed our understanding of systemic lupus erythematosus pathogenesis. Recent advances reveal that metabolic reprogramming and immune dysregulation engage in an intricate bidirectional relationship that actively drives disease progression. Immune cell activation triggers profound metabolic alterations characterized by enhanced nutrient uptake and comprehensive remodeling of energy metabolic pathways. These metabolic adaptations subsequently exert significant influence on immune cell differentiation and functional states, establishing a self-amplifying feedback loop that perpetuates autoimmune responses [98, 99].

While current SLE therapeutics primarily target immunological pathways, emerging research demonstrates that targeting immunometabolic pathways offers a novel paradigm for restoring immune homeostasis. This innovative approach focuses on precise modulation of cellular metabolic states while circumventing the limitations associated with conventional broad-spectrum immunosuppression [100]. Within this evolving therapeutic landscape, Traditional Chinese Medicine demonstrates distinctive potential through its unique capacity for multi-target regulation that simultaneously addresses both immunological and metabolic dimensions of SLE pathology, positioning it as a promising complementary approach to current treatment strategies.

### Direct organ protection

#### Renal protection through anti-fibrotic mechanism

As a systemic autoimmune disorder, SLE typically manifests with multi-organ damage, with renal impairment representing one of the most clinically significant complications. In lupus nephritis, renal fibrosis emerges as a pivotal determinant of poor long-term prognosis [101]. The characteristic pathological features include immune complex deposition along tubular basement membranes, accompanied by tubulointerstitial immune cell infiltration and progressive interstitial fibrosis. The binding of anti-dsDNA antibodies to renal cellular components, particularly proximal tubular epithelial cells, initiates cascades of renal inflammation and fibrotic transformation. Beyond these established mechanisms, epigenetic regulation encompassing DNA methylation patterns, histone modifications, and microRNA networks is increasingly recognized as a crucial contributor to renal fibrosis pathogenesis [102].

TCM formulations demonstrate distinctive efficacy in counteracting renal fibrotic processes and improving clinical outcomes. The JP exhibits comprehensive renoprotective properties in MRL/lpr mice, significantly ameliorating characteristic pathological alterations, including mesangial hyperplasia, basement membrane thickening, and immune complex deposition [103]. These protective effects correlate with multi-target inhibition of renal fibrotic signaling cascades. Mechanistic studies reveal that JP orchestrates a sophisticated anti-fibrotic program through coordinated downregulation of α-smooth muscle actin (α-SMA) and transforming growth factor β1 (TGF-β1) expression, while simultaneously enhancing farnesoid X receptor transcriptional activity. This multifaceted regulation effectively disrupts the epithelial-mesenchymal transition cascade, which represents a central pathway in renal fibrosis development [85].

Additional TCM components contribute complementary anti-fibrotic mechanisms through distinct molecular pathways. Demethylzeylasteral demonstrates notable efficacy in alleviating podocyte injury and subsequent fibrotic progression in MRL/lpr mice through concurrent suppression of inflammatory signaling and enhancement of autophagic flux via inhibition of the IL-17A/JAK2-STAT3 pathway [104]. Similarly, Cordyceps proteins modulate the STAT3/mTOR/NF-κB signaling network in lupus nephritis models, resulting in reduced proteinuria, diminished renal inflammatory infiltration, and attenuated fibrosis development [105]. These findings collectively underscore the multi-targeted approach of TCM in addressing the complex pathophysiology of renal complications in SLE.

#### Cellular protection and regeneration

Dysregulated programmed cell death signaling constitutes a fundamental component of SLE immunopathogenesis. The aberrant accumulation of cellular components and impaired clearance of cellular debris, particularly nucleic acids and nucleic acid-protein complexes, generate a persistent source of autoantigens. These autoantigens subsequently activate autoreactive B cell responses and promote type I interferon production, thereby establishing a vicious cycle that drives disease progression [106]. Enhanced apoptotic signaling has been consistently detected in T lymphocytes from SLE patients and demonstrates significant correlation with disease activity metrics, providing a potential biomarker for monitoring therapeutic response [107].

Pathological cell death patterns extend to multiple cellular populations, creating a complex network of cellular dysfunction that includes neutrophils [108], monocytes [109], glomerular parenchymal cells, renal tubular epithelial cells, and renal interstitial inflammatory components [110]. Multiple programmed cell death modalities contribute collectively to SLE pathology, including autophagy, ferroptosis, pyroptosis, NETosis, necroptosis, apoptosis, and the recently characterized PANoptosis. Understanding the intricate interconnections among these diverse cell death pathways may reveal novel therapeutic opportunities for SLE intervention and management.

TCM interventions target specific cell death pathways with remarkable precision, offering multiple strategies for cellular protection and regeneration. JP promotes Bim-mediated apoptosis in double-negative T cells through suppression of UBE2M expression and subsequent CRL-dependent Bim degradation, thereby restoring immune balance [39]. Curcumin, a principal active component derived from turmeric rhizomes, enhances double-negative T cell apoptosis in both genetic and induced SLE models, ultimately reducing pathological T cell accumulation and ameliorating disease manifestations through modulation of critical signaling pathways [111]**.**

Total glucosides of paeony, extracted from Paeonia lactiflora roots, suppress PANoptosis [112] activation in podocytes of MRL/lpr mice through modulation of the STAT2-ZBP1 axis, effectively preserving renal structural integrity and function [113]. Celastrol, a bioactive diterpenoid from Tripterygium wilfordii, selectively induces apoptosis in CD138^+^ T cell populations, thereby limiting the accumulation of autoimmune T cells and preventing disease progression in murine lupus models [114]. Furthermore, quercetin demonstrates multiple beneficial bioactivities, including antimicrobial, antioxidant, and anti-inflammatory properties, while also modulating aged CD4^+^ T cell populations through suppression of T follicular helper cell differentiation and enhanced apoptotic clearance, thereby effectively eliminating immunosenescent phenotypes and alleviating SLE pathology [115].

### Targeting immunometabolism: correcting metabolic reprogramming in SLE

**Fig. 4:**
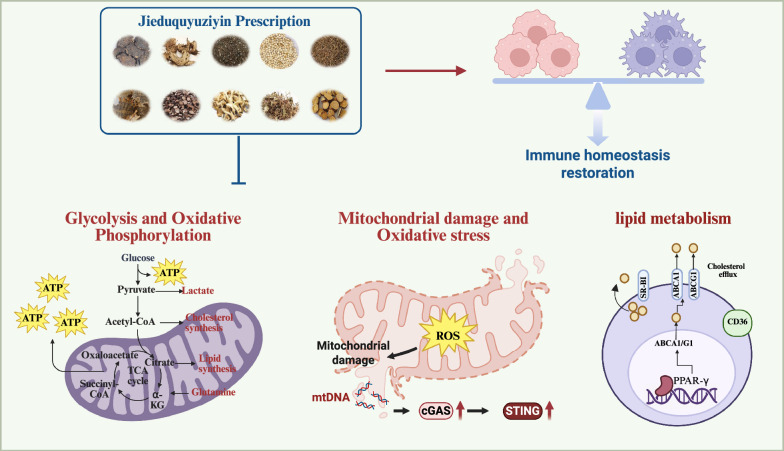
Jieduquyuziyin prescription (JP) ameliorates SLE by counteracting metabolic dysregulation. Schematic representation of JP rectifying metabolic perturbations in SLE, including aberrant glycolysis, oxidative phosphorylation, mitochondrial dysfunction, and disordered lipid metabolism

Cellular metabolic pathways in immune cells are dynamically regulated by microenvironmental signals and cellular demands, creating essential connections between metabolic activity and immunological function. These interconnected pathways include glycolysis, the pentose phosphate pathway, the tricarboxylic acid cycle, oxidative phosphorylation, and complex lipid and amino acid metabolic networks that collectively determine immune cell fate and function [116]. The emerging understanding of these metabolic-immunological connections provides new opportunities for therapeutic intervention in SLE **(**Fig. 4**).**

#### Regulation of glycolysis and oxidative phosphorylation

Immune cells in SLE consistently demonstrate enhanced glycolytic flux, frequently adopting a Warburg-like metabolic profile even under normoxic conditions to meet the biosynthetic and energetic demands of persistent activation. This metabolic reprogramming spans multiple immune cell types, including T lymphocytes, B cells, monocytes, macrophages, and neutrophils, creating a systemic metabolic disturbance that perpetuates autoimmune responses [117].

CD4^+^ T cells from SLE patients exemplify this metabolic shift, displaying significantly enhanced glycolytic capacity and oxidative phosphorylation activity that correlates with disease severity [118]. This metabolic alteration is coordinated through multiple interconnected signaling pathways, including mTOR pathway hyperactivation [119], HIF-1α stabilization [120], and upregulated glucose transporter Type 1 (GLUT1) expression [121], which collectively drive the pathological metabolic reprogramming observed in SLE.

JP ameliorates SLE pathology partially through normalization of aberrant T cell glycolytic metabolism. Comprehensive metabolic analysis reveals that treatment significantly reduces levels of glycolysis-related metabolites and enzymatic components in CD4^+^ T cells, including glucose, pyruvate, lactate, GLUT1, hexokinase 2, pyruvate kinase M2, and lactate dehydrogenase A, thereby restoring metabolic balance [73]. In B cell compartments, elevated BAFF signaling promotes aerobic glycolytic programming that supports pathological proliferation and antibody production [122], while monocarboxylate transporter 1 further regulates B cell activation through modulation of pyruvate uptake and glycolytic flux [123].

Additionally, TLR9 activation via CpG stimulation combined with IFNα signaling induces metabolic switching to glycolysis in CD27^+^ IgD^+^ unswitched memory B cells, driving their differentiation toward CD27^hi^ CD38^hi^ plasmablast phenotypes that contribute to autoantibody production [124]. Importantly, JP administration suppresses this glycolysis-dependent B cell activation through the AMPK/PKM2 pathway, reducing germinal center responses and effector B cell generation, thereby attenuating disease progression through precise metabolic modulation [80].

#### Restoration of mitochondrial function and attenuation of oxidative stress

Mitochondrial dysfunction refers to impaired mitochondrial activity, resulting in insufficient energy production, increased generation of reactive oxygen species, and formation of superoxide anion radicals. The synthesis of reactive oxygen species and heightened oxidative stress damage mitochondrial components, including lipids, proteins, and mtDNA [125]. Mitochondrial dysfunction refers to impaired mitochondrial activity, resulting in insufficient energy production, increased generation of reactive oxygen species, and formation of superoxide anion radicals. The synthesis of reactive oxygen species and heightened oxidative stress damage mitochondrial components, including lipids, proteins, and mtDNA. Mitochondrial dysfunction represents a cornerstone of immunometabolic abnormalities in SLE pathogenesis [100]. T cells and neutrophils from patients consistently demonstrate elevated mitochondrial membrane potential, disrupted calcium homeostasis, excessive reactive oxygen species production, and impaired mitophagic clearance [126]. Particularly in neutrophils, mitochondrial reactive oxygen species generation promotes extracellular release of oxidized mitochondrial DNA, which subsequently activates the cGAS-STING pathway and amplifies type I interferon responses, thereby establishing a persistent proinflammatory feedback loop that drives disease activity [126].

Traditional Chinese Medicine provides multi-target therapeutic strategies for mitochondrial functional restoration that address these complex abnormalities. JP significantly attenuates renal oxidative stress damage through multiple complementary mechanisms, including inhibition of reactive oxygen species and malondialdehyde accumulation, restoration of mitochondrial membrane potential stability, and enhancement of superoxide dismutase antioxidant activity. These combined effects augment glucocorticoid efficacy while reducing treatment-associated side effects, representing a valuable complementary approach to conventional therapy [93].

Furthermore, JP may ameliorate oxidative stress-mediated renal injury by delaying proteasomal degradation of p53, potentially limiting Nicotinamide Adenine Dinucleotide Phosphate (NADPH) oxidase substrate availability and reducing renal reactive oxygen species production through modulation of the G6PD/NOX2/4-ROS-MAPK signaling network [127]. The flavonoid compound rutin demonstrates additional efficacy in ameliorating T cell oxidative stress, potentially through PPARγ/NF-κB/STAT3 pathway modulation, which suppresses proinflammatory cytokine secretion, improves hematological parameters and renal histopathological manifestations, and alleviates lupus-like symptomatic presentation [128].

Luteolin administration provides another therapeutic approach by reducing HIF-1α expression and oxidative stress in macrophage populations, thereby mitigating renal injury mediated by infiltrating macrophages in MRL/lpr mice and ameliorating lupus nephritis manifestations through modulation of the renal microenvironment [44]. These multi-targeted approaches demonstrate how TCM can simultaneously address multiple aspects of mitochondrial dysfunction and oxidative stress in SLE.

#### Remodeling metabolite networks

In the evolving paradigm of SLE pathogenesis, metabolic reprogramming, with a particular focus on lipid networks, has emerged as a critical regulator of autoimmune dysregulation that extends beyond the classical roles of lipids in structural integrity and energy storage. SLE patients frequently exhibit pronounced dyslipidemia, characterized by reduced high-density lipoprotein cholesterol, elevated triglycerides, and increased low-density lipoprotein, a pattern that correlates with disease severity and elevated cardiovascular risk, a leading cause of morbidity in this population [129]. These alterations are not merely secondary phenomena but actively contribute to immune dysfunction, as diverse lipid species modulate key cellular processes including proliferation, differentiation, and survival signaling, thereby directly shaping autoimmune responses [130]. Experimental evidence reveals that splenic fibroblastic reticular cell-derived acetylcholine activates lipid metabolic pathways that stimulate autoimmune B cell responses, while pharmacological inhibition of fatty acid oxidation or genetic deletion of CD36 in B cells suppresses autoreactive B cell activation and autoantibody production in lupus models [131]. This mechanistic understanding is further supported by the beneficial effects of conventional lipid-lowering agents such as statins in SLE management, highlighting lipid metabolism as a promising therapeutic target [132]. Within this framework, the traditional formulation JP has demonstrated efficacy in ameliorating lipid metabolic abnormalities in MRL/lpr mice by stabilizing body weight, attenuating aortic plaque formation, and enhancing the expression of cholesterol efflux mediators, including ATP-binding cassette transporters A1 and G1, scavenger receptor class B type I, and peroxisome proliferator-activated receptor gamma, thereby reducing foam cell formation in macrophages and re-establishing cholesterol homeostasis [40]. Further mechanistic dissection of JP’s multi-component formulation shows that artesunate, one of its active constituents, counteracts SLE-accelerated atherosclerosis through restoration of PPARγ-mediated cholesterol efflux and disruption of lipid raft-organized TLR9/MyD88 signaling [133], while alisol B 23-acetate, derived from Alisma orientale, improves lipid metabolic parameters in SLE models via modulation of CD36 signaling pathways [42]. Advanced metabolomic analyses further corroborate that JP exerts systemic therapeutic effects by remodeling unsaturated fatty acid metabolism and phospholipid metabolic networks [134, 135]. Collectively, these findings illustrate a shift in therapeutic strategy from broad immunosuppression toward precision metabolic correction. By concurrently targeting glucose metabolism, mitochondrial function, oxidative stress, and specific metabolite networks, emerging approaches seek to therapeutically reprogram hyperactive immunity and restore metabolic and functional homeostasis. However, a central challenge remains the translation of these sophisticated preclinical insights into individualized, clinically actionable treatment regimens.

Future progress will require leveraging advanced metabolomic technologies to develop validated biomarkers for guiding therapeutic decisions and objectively evaluating treatment efficacy, while also addressing the complexity of metabolic networks in different patient subsets. Additionally, research should focus on establishing clinically applicable metabolic profiling that can predict treatment response and monitor therapeutic effectiveness in SLE patients, ultimately enabling personalized medicine approaches. As our understanding of immunometabolism continues to evolve, integrating metabolic interventions with conventional and emerging immunotherapies may offer new opportunities for achieving sustained disease control and improving long-term outcomes for patients with this complex autoimmune disorder. The multi-target, system-level approach of Traditional Chinese Medicine, with its ability to simultaneously address multiple metabolic and immunological abnormalities, represents a particularly promising direction for future therapeutic development in SLE management.

## TCM in the treatment of SLE via the “gut-microbiota-metabolism” axis

The gastrointestinal tract, with its vast surface area, serves not only as the primary site for nutrient digestion and absorption but also constitutes a crucial part of the body’s immune system, housing a large and complex population of immune cells. It forms a dynamic micro-ecosystem that continuously interacts with the external environment [136]. Accumulating evidence reveals significant alterations in the gut microbiota composition of patients with SLE compared to healthy individuals. These dysbiotic changes are characterized by a relative reduction in beneficial bacteria, such as those within the phylum Firmicutes, concomitant with an overgrowth of potentially pathogenic bacteria like Proteobacteria, alongside an overall decrease in microbial diversity [137]. More specifically, patients with active SLE exhibit marked increases in the abundance of specific microbiota, including *Streptococcus anginosus*, *Megasphaera*, *Fusobacterium*, *Veillonella*, *Geobacter*, *Odoribacter*, and *Blautia* within the *Lachnospiraceae* family [138, 139].

The structure and function of the gut microbiota are modulated by a combination of genetic predisposition, environmental factors, and dietary habits. Among these, diet is considered one of the most malleable key factors, as ingested nutrients directly provide substrates for the growth and metabolism of specific microbial populations, thereby influencing the overall ecological balance of the gut community [140].

In this context, orally administered TCM, whose active components undergo intestinal absorption and microbial metabolism, can exert regulatory effects on the pathological state of SLE. Growing research suggests that TCM may ameliorate SLE-related aberrant immune responses systematically by remodeling the gut microbiota structure, subsequently modulating the function and population of immune cells, promoting the development and repair of the intestinal mucosal barrier, and enhancing gut immune defense capabilities [141]. This mechanistic framework provides a theoretical basis for understanding the role of the “Gut-Microbiota-Metabolism” axis in SLE treatment and opens new avenues for explaining the immunomodulatory effects of TCM.

### Remodeling the gut ecology: from structure to barrier integrity

The gut microbiota plays a pivotal role in maintaining the structural and functional integrity of the intestinal barrier. An intact intestinal barrier effectively prevents the translocation of luminal pathogens and harmful metabolites into the systemic circulation, thereby averting systemic immune activation [142]. Dysbiosis can lead to the downregulation of intestinal epithelial tight junction protein expression, increased mucosal permeability, and the onset of “leaky gut” [143]. Under these conditions, pathogen-associated molecular patterns such as bacterial lipopolysaccharide (LPS) translocate into the bloodstream, promoting the production of pro-inflammatory cytokines like type I interferons through the activation of TLR signaling pathways (e.g., TLR4), directly exacerbating SLE disease activity [144]. TLR9 activation has been shown to induce Paneth cell degranulation in the small intestine, promoting the release of antimicrobial peptides, whereas TLR9 deficiency can lead to Paneth cell abnormalities and commensal dysbiosis. Administration of *Lactobacillus johnsonii N6.2* was found to increase IFN expression and expand Paneth cell numbers in diabetes-prone rats via upregulation of TLR9, inducing immune perturbations [145]**.**

Research has discovered that the JP not only effectively reduces SLE disease activity but also decreases the dosage requirements of glucocorticoids and their associated adverse effects. This mechanism may be related to its specific modulation of the gut microbiota, such as promoting the proliferation of beneficial bacteria, including *Akkermansia, Parasutterella,* and *Alistipes *[146]. Notably, *Akkermansia muciniphila* and *Parabacteroides distasonis* have been shown to act synergistically by promoting intestinal Group 3 Innate Lymphoid Cells (ILC3s), thereby helping to prevent colitis [147], improving intestinal barrier function, and enhancing mucus layer thickness. Concurrently, multiple studies have found that JP can effectively target TLR family members, such as TLR7 and TLR9, thereby ameliorating disease progression [40, 84]. These findings indicate that JP may counteract the negative impact of glucocorticoids on the gut microecology by enriching key beneficial bacteria with barrier-repairing and immunomodulatory functions, thus providing therapeutic benefits for SLE while mitigating hormonal side effects.

### Microbiota-derived metabolites: key messengers linking the gut and systemic immunity

Through the fermentation of dietary fibers or the transformation of host-derived substances, the gut microbiota produces a spectrum of bioactive metabolites. These molecules serve as crucial mediators in the cross-talk between the microbiota and the host immune system [142]. They can enter the circulatory system and exert long-distance regulatory effects on the immune and inflammatory status of distal organs, such as the kidneys, skin, and joints. The gut microbiome interacts with host immune cells and metabolic pathways, modulating both innate and adaptive immune responses [148].

#### Immunomodulatory roles of short-chain fatty acids

Among gut microbiota metabolites, Short-Chain Fatty Acids (SCFAs) hold particular importance. Primarily produced by microbial fermentation of dietary fibers, SCFAs are among the most abundant metabolites in the gastrointestinal tract [149]. Butyrate, as a representative SCFA, possesses anti-inflammatory and antioxidant properties, and its therapeutic potential has been extensively documented [150]. SCFAs regulate immune cell metabolism through multiple mechanisms, including metabolic assimilation, acetyl-CoA production, G protein-coupled receptor (GPCR) signaling, and histone deacetylase (HDAC) inhibition, thereby influencing immune cell function and differentiation [151].

The inhibition of HDAC in peripheral T cells mediated by SCFAs promotes anti-inflammatory effects by expanding and supporting the differentiation of regulatory T (Treg) cells under various conditions [152]. The potential of SCFA intake (butyrate and acetate) to avert hypertension has been demonstrated, specifically by restoring the interactions between the gut, immune system, and vascular wall to counteract SLE manifestations induced by TLR7 activation [153].

TCM compounds are rich in polysaccharides. Studies indicate that natural polysaccharides can serve as carbon sources for gut microbiota, increasing microbial diversity, modulating its composition, and consequently improving disease outcomes [154]. More specifically, polysaccharides can modulate gut microbiota composition, stimulate SCFA production, and mediate the reduction of inflammation [155]. For instance, Astragalus polysaccharides have been shown to alleviate ulcerative colitis by restoring SCFA production and modulating Th17/Treg cell homeostasis in a microbiota-dependent manner [156]. Polysaccharides from *Hirsutella sinensis* (related to *Cordyceps sinensis*), previously found to exert anti-inflammatory, anti-diabetic, and anti-obesity effects in high-fat diet-fed mice by modulating gut microbiota and increasing the abundance of commensal Parabacteroides, have now been demonstrated to ameliorate disease severity in an imiquimod-induced SLE mouse model. Improvements included reductions in spleen weight, proteinuria, serum anti-dsDNA autoantibodies, and signal transducer and activator of transcription 4 (STAT4) levels [157].

High-fiber diets have been shown to improve metabolic and allergic diseases primarily through microbiota-dependent fermentation to SCFAs. These SCFAs promote tissue barrier integrity, mucus production, IgA secretion, and Treg cell differentiation, thereby supporting an anti-inflammatory environment [158]. Consistently, studies using female NZB/W F1 (SLE-prone) mice demonstrated that treatment with resistant starch (RS) or inulin-type fructans (ITF), particularly RS, prevented the development of lupus nephritis and renal damage, and improved acetylcholine-induced aortic relaxation and vascular oxidative stress [159].

#### Signaling pathway regulation by tryptophan metabolites

Tryptophan metabolism exemplifies host-microbiota symbiosis, involving three competitive pathways: the host cell kynurenine pathway, the microbial indole pathway, and the host serotonin pathway. In SLE, the metabolic balance often shifts towards the kynurenine pathway, resulting in the accumulation of neurotoxic and pro-inflammatory kynurenine metabolites, while the microbial indole pathway is frequently suppressed. Microbiota-derived molecules such as indole-3-propionic acid and indole-3-aldehyde act as endogenous ligands for the Aryl Hydrocarbon Receptor (AhR). AhR activation not only induces interleukin-22 (IL-22) production in intestinal epithelial cells, promoting barrier repair, but also directly suppresses the pathogenicity of Th17 cells and drives Treg cell differentiation, thereby precisely regulating immune balance [100].

The gut microbiota metabolizes tryptophan and other amino acids into bioactive compounds like indole and indoleacrylic acid [160]. The gut microbe-mediated tryptophan-indolepyruvate metabolic pathway plays significant roles in both physiological and pathological processes and is a focus of dietary strategies for preventing and treating metabolic diseases. These metabolites possess antioxidant, anti-inflammatory, and immunomodulatory properties. In SLE, they may alleviate the disease by enhancing intestinal barrier function, modulating immune cell activity, and reducing inflammatory responses [161].

Berberine (BBR), a primary active component of the TCM herb *Coptis chinensis*, has been shown to treat DSS-induced colitis in rats by modulating gut microbiota-associated tryptophan metabolites to activate AhR, which significantly improves compromised intestinal barrier function [162]. Furthermore, BBR treatment markedly alters the levels of tryptophan metabolites, including indole, indole-3-acetamide, indole-3-carboxaldehyde, indole-3-pyruvic acid, and indole-3-acetic acid [163]. BBR has also demonstrated therapeutic potential in SLE, effectively inhibiting inflammatory cytokine expression and reducing the secretion of pro-inflammatory markers [164]. Although direct evidence linking BBR’s action in SLE specifically to tryptophan metabolism modulation is currently lacking, it is a plausible mechanism worthy of further investigation.

#### Bile acid metabolism and inflammatory balance

Bile acid metabolism forms a critical bridge connecting the liver and the gut, with the microbiota acting as key “transformers.” Primary bile acids synthesized in the liver are converted into secondary bile acids by gut bacteria (e.g., *Bacteroides*, *Clostridium*). Both primary and secondary bile acids function as signaling molecules, activating the Farnesoid X Receptor (FXR) and the G Protein-Coupled Bile Acid Receptor 5 (TGR5) [165, 166].

FXR is a key mediator in the cross-talk between bile acid metabolism and the gut microbiome. FXR activation exerts broad anti-inflammatory effects: it inhibits the NLRP3 inflammasome in macrophages [167]; suppresses dendritic cell maturation, thereby attenuating the presentation of autoantigens [168]; and regulates glucose and lipid metabolism homeostasis in the liver [169]. FXR also plays a significant role in SLE patients. Genetic deletion of FXR led to impaired T follicular helper (Tfh) cell differentiation, dysfunctional germinal center (GC) responses, and limited antibody production. Consequently, both T cell-specific FXR deficiency and pharmacological inhibition of FXR signaling by Ursodeoxycholic Acid (UDCA) prevented lupus-like disease in mice. Mechanistically, FXR intrinsically regulates HMGCR-mediated cholesterol biosynthesis and NPC2-mediated lysosomal cholesterol transport in Tfh cells, thereby sequentially controlling Tfh cell proliferation [170].

**Fig. 5:**
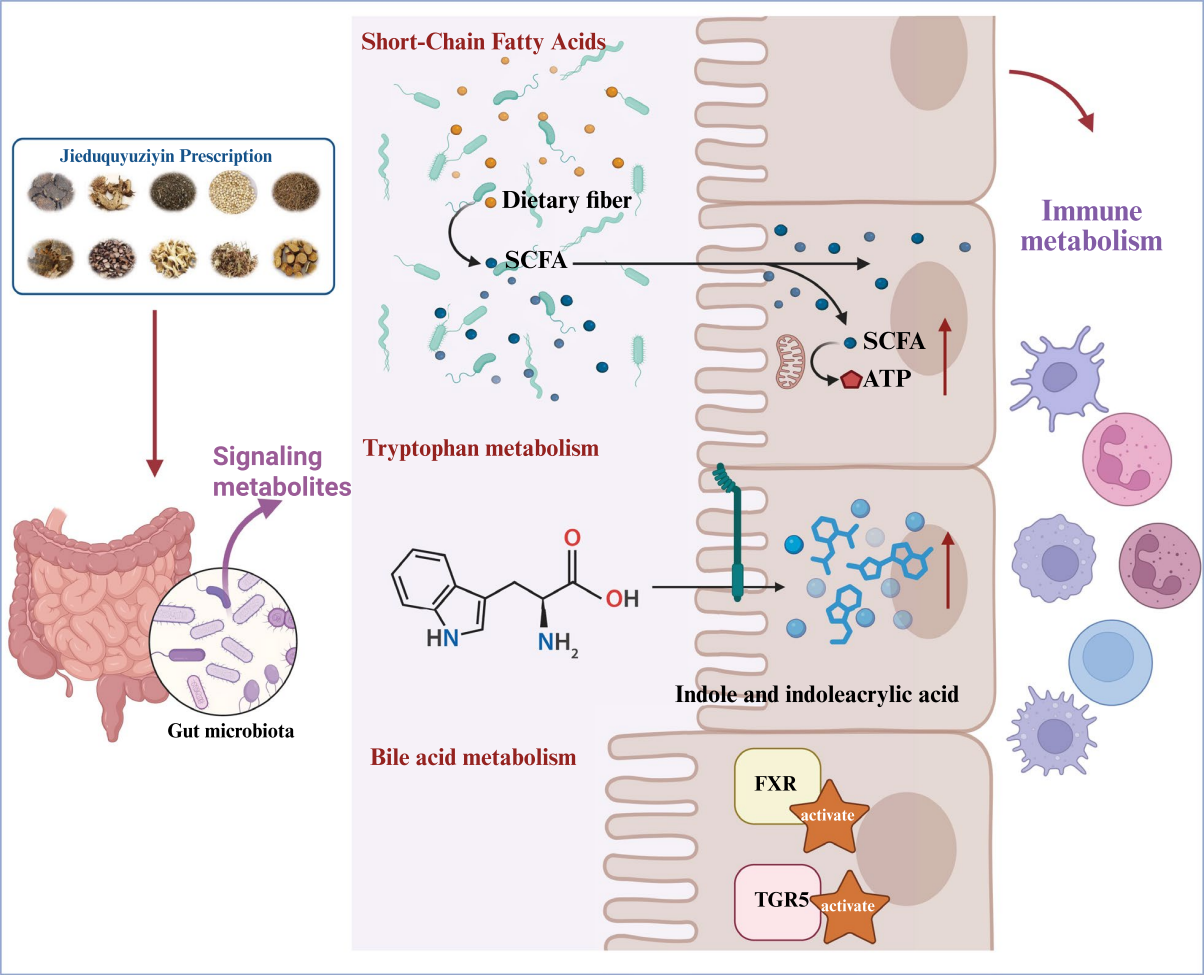
Jieduquyuziyin prescription (JP) alleviates SLE via modulation of gut microbiota-derived metabolites. Schematic diagram illustrating how JP ameliorates SLE by reshaping gut microbiota composition and function, thereby influencing key microbial metabolic pathways, including short-chain fatty acid (SCFA) production, tryptophan metabolism, and bile acid metabolism

SLE patients often exhibit an abnormal bile acid pool composition, which reciprocally interacts with gut dysbiosis. Research has found that JP can significantly increase FXR expression and inhibit its downstream targets, namely NF-κB and α-SMA. Overall, JP may mediate the activation of renal FXR expression, suppress NF-κB and α-SMA, exerting anti-inflammatory and anti-fibrotic effects in the prevention and treatment of LN, thereby improving its pathological progression [85]** (**Fig. 5**).**

In summary, a growing body of evidence underscores the critical role of the gut-microbiota-metabolism axis in the pathogenesis of Systemic Lupus Erythematosus. Traditional Chinese Medicine emerges as a promising multi-targeted therapeutic approach, capable of ameliorating SLE by restoring gut ecological balance, modulating microbiota-derived metabolites including SCFAs, tryptophan derivatives, and bile acids, and subsequently recalibrating host immune homeostasis. This integrative strategy not only highlights the potential of harnessing host-microbe interactions for treatment but also bridges traditional knowledge with modern immunometabolic science, paving the way for novel therapeutic paradigms in autoimmune disease management.

## Challenges and future perspectives

SLE remains a formidable clinical challenge, often termed an “incurable cancer” due to its refractory nature and severe complications such as lupus nephritis, neuropsychiatric involvement, and cardiovascular disease. The rising global prevalence of SLE, combined with the high toxicity and substantial economic burden associated with conventional therapies, underscores the urgent need for safer and more effective treatment strategies. For centuries, TCM has been used to alleviate clinical symptoms in SLE patients and mitigate the adverse effects of glucocorticoids and other immunosuppressive agents. The concept of “enhancing efficacy and reducing toxicity” through TCM has been consistently observed in long-term clinical practice. However, despite this promising potential, several scientific and translational barriers must be overcome to fully integrate TCM into contemporary SLE management.

The multi-component, multi-target nature of TCM formulations presents both an advantage and a challenge. While network pharmacology and omics technologies have begun to map these complex interactions, a deeper understanding of their mechanisms requires validation using advanced tools such as genetically engineered animal models, single-cell sequencing, and spatial transcriptomics. These approaches will help delineate the molecular circuitry through which TCM modulates immune and metabolic pathways in SLE. Additionally, variability in herbal sources, processing methods, and formulation consistency poses significant obstacles to reproducibility and dosing accuracy. Future efforts should prioritize the standardization of extraction protocols, the establishment of biomarker-based quality control, and the implementation of Good Manufacturing Practice guidelines for TCM products.

Clinical translation also faces challenges. Although many studies support the efficacy of TCM, there is a need for more rigorously designed randomized controlled trials with larger sample sizes, long-term follow-up, and integrated omics endpoints. Such trials are essential to validate TCM’s steroid-sparing, organ-protective, and immunometabolic effects. Furthermore, personalized TCM strategies that incorporate both syndrome differentiation and molecular profiling represent a promising direction for future research. Another underexplored area is the potential synergy between TCM and emerging biologic or cell-based therapies such as CAR-T. Investigating whether TCM can enhance the efficacy or reduce the toxicity of these targeted treatments, particularly in refractory SLE, could open new therapeutic avenues.

The role of the gut-microbiota-immunity axis in SLE has garnered increasing attention. While TCM has shown compelling effects on gut microbiota composition, most evidence remains correlative. Establishing causality will require mechanistic studies using germ-free models, fecal microbiota transplantation, and metabolite tracing techniques. Finally, to achieve global recognition, TCM must meet international regulatory standards for safety, efficacy, and quality. Collaborative initiatives among TCM researchers, immunologists, and regulatory bodies are critical to developing evidence-based guidelines and facilitating worldwide acceptance.

In summary, integrating TCM into SLE care represents a shift from single-target suppression toward system-level immune recalibration. By addressing immunometabolic dysregulation, epigenetic remodeling, and host-microbiota interactions, TCM offers a holistic and personalized approach aligned with the principles of precision medicine. Future research should leverage multi-omics, artificial intelligence, and advanced bioinformatics to decode TCM’s systemic effects, validate its clinical utility, and ultimately transform SLE treatment through integrative and sustainable therapeutic strategies.

## Conclusion

This article systematically elucidates the fundamental pathology of SLE as a complex autoimmune disorder, characterized by immune imbalance and multi-organ damage, while critically highlighting the limitations of conventional immunosuppressive therapies. In this context, it rigorously demonstrates the distinctive value of TCM, exemplified by the herbal formulation JP, in re-establishing immune homeostasis through multi-targeted, systemic interventions. The study illustrates how JP not only harmonizes adaptive and innate immunity by correcting the Th17/Treg imbalance and suppressing pathogenic B cells, but also precisely modulates immunometabolic reprogramming, reversing hyperactive glycolysis and restoring mitochondrial function. Additionally, through the emerging “gut-microbiota-metabolism-immunity” axis, JP remodels gut ecology and regulates microbial metabolites, such as short-chain fatty acids, tryptophan derivatives, and bile acids, thereby exerting synergistic and enhanced effects in organ protection and systemic immune regulation. This work integrates the holistic principles of TCM with modern systems biology and immunometabolism, providing a robust scientific foundation for the application of TCM in treating SLE and underscoring its potential significance.

## Data Availability

No datasets were generated or analysed during the current study.

## Abbreviations

AhR: Aryl hydrocarbon receptor
AKT: Protein kinase B
AMPK: AMP-activated protein kinase
BBR: Berberine
CVD: Cardiovascular disease
CAR: Chimeric antigen receptor
DNT: Double-negative T-cell
FXR: Farnesoid X receptor
GC: Germinal center
GLUT1: Glucose transporter type 1
GPCR: G protein-coupled receptor
HDAC: Histone deacetylase
ILC3s: Group 3 innate lymphoid cells
IL-22: Interleukin-22
JP: Jieduquyuziyin prescription
ITF: Inulin-type fructans
LN: Lupus nephritis
LPS: Lipopolysaccharide
mTOR: Mammalian target of rapamycin
NADPH: Nicotinamide adenine dinucleotide phosphate
NETs: Neutrophil extracellular traps
NF-κB: Nuclear factor kappa-B
NLR: Neutrophil-to-lymphocyte ratio
NPSLE: Neuropsychiatric SLE
Nrf2: Nuclear factor erythroid 2-related factor 2
PI3K: Phosphoinositide 3-kinase
PLR: Platelet-to-lymphocyte ratio
RS: Resistant starch
SCFAs: Short-chain fatty acids
SLE: Systemic lupus erythematosus
SLEDAI: SLE disease activity index
STAT4: Signal transducer and activator of transcription 4
TCM: Traditional Chinese medicine
Tfh: T follicular helper
TGF-β1: Transforming growth factor β1
TGR5: The G protein-coupled bile acid receptor 5
TLR: Toll-like receptor
Treg: Regulatory T
UDCA: Ursodeoxycholic acid
WM: Western medicine
α-SMA: α-Smooth muscle actin
